# COVID-19 pandemic: influence of relationship status on stress, anxiety, and depression in Canada

**DOI:** 10.1017/ipm.2021.1

**Published:** 2021-01-14

**Authors:** Nnamdi Nkire, Izu Nwachukwu, Reham Shalaby, Marianne Hrabok, Wesley Vuong, April Gusnowski, Shireen Surood, Andrew J. Greenshaw, Vincent I. O. Agyapong

**Affiliations:** 1 Department of Psychiatry, Faculty of Medicine and Dentistry, University of Alberta, Edmonton, Canada; 2 Addiction and Mental Health, Alberta Health Services, Edmonton, Canada; 3 Department of Psychiatry, Cumming School of Medicine, University of Calgary, Calgary, Canada

**Keywords:** Anxiety, COVID-19, depression, e-mental health, mobile phones, pandemic, relationship status, stress, text, Text4Hope

## Abstract

**Objective:**

To examine the impact of relationship status on levels of stress, anxiety, and depression during the coronavirus (COVID-19) pandemic to identify relationship status groups who are at greater risk of mental health difficulties.

**Methods:**

The sample was drawn from individuals who subscribed to the Text4Hope program, a cognitive behavioral therapy inspired text messaging service developed to support Albertans during the COVID-19 pandemic. A survey link was sent to the subscribers to ascertain their relationship status and assess psychopathology using the Perceived Stress Scale-10 (PSS-10), Generalized Anxiety Disorder 7-item (GAD-7) scale, and Patient Health Questionnaire-9 (PHQ-9). Data analysis was carried out using SPSS-26 for descriptive statistics.

**Results:**

Within the first 6 weeks of the pandemic, 8267 of 44·992 subscribers responded to the online survey giving a response rate of 19.4%. Mean scores on the PSS, GAD-7, and PHQ-9 were highest among those who were single and lowest among those who were widowed. Overall, mean scores on the PHQ-9 were higher in groups who self-identified as separated or divorced when compared with groups who identified as having partners, including the categories of married or cohabiting.

**Conclusions:**

Relationship status during the COVID-19 pandemic has an influence on the mental health of individuals. Our findings highlight relationship groups at risk of mental health problems during the pandemic and for whom treatments and mitigation should be targeted.

## Introduction

### Background

Since its discovery in Wuhan China, coronavirus disease-19 (COVID-19) has spread rapidly across the world in a few months (Oud *et al.* 2019). Having been declared a global pandemic in January 2020, Canada has not been spared (Brown *et al.* 2011). As it has progressed, the pandemic leaves in its wake a significant death toll, worsening economic indices, and increased global disease burden (Gautam & Sharma, 2020). This trail of devastation has caused governments to take unprecedented actions to curb the spread of this novel disease, including partial shutdown of the economy, physical distancing, and quarantine (Abba-Aji *et al.* 2020; Nwachukwu *et al.* 2020). These measures coupled with the direct effects of the illness itself have disturbed the natural rhythm and structure of individuals’ lives, which in turn has had an adverse impact on mental health and well-being (Wang *et al.* 2020).

Quarantine and self-isolation are well-established means for managing highly contagious disease outbreaks in an epidemic (Taha *et al.* 2014). The two terms, although used interchangeably, have different meanings. Self-isolation is the sequestration of individuals who have been diagnosed with a contagious disease from those who are not sick (Brooks *et al.* 2020, Manuell & Cukor, 2010), while quarantine is the separation and limitation of movement of individuals who have potentially been exposed to a contagious disease to see whether they become unwell, thereby reducing the risk of infection to others (Williams & Potts, 2010; Taha *et al.* 2014). These well-intentioned methods of managing a pandemic produce unintended consequences. Prior research indicates that both measures may increase anxiety, stress and depression (Nkire *et al.* 2021), increase suicidal risk (Barbisch *et al.* 2015), escalate boredom, increase fears of infecting family particularly among those with young children, limit supplies of essential goods, affect family finances, induce frustration and anger and litigation (Miles, 2015; Brooks *et al.* 2020), and in some circumstances, result in the stigmatization of affected individuals. As well, by putting cohabiting individuals (eg. partners, roommates and families) in unusually close proximity for a long time in a mostly closed unit, it may expose or worsen existing tensions in relationships. While for single individuals, it may reduce access to previous supports, which may in turn increase their stress.

Stress levels, anxiety, and depression are usually elevated during crises (Wang *et al.* 2020). As work places have closed and individuals have become limited to their family units or households, intimate partner relationships became an important source of coping with stress posed by the pandemic. Relationships have a bidirectional association with mental health such that a good relationship bodes well for good mental health, while severe mental illness may pose a strain on relationships. Some researchers have observed that relationships on the whole predict better mental health outcomes (Braithwaite & Holt-Lunstad, 2017). They also note that the quality of the relationship plays a role in ensuring good mental health. They further posit that established committed relationships are associated with greater benefit to mental health (Braithwaithe & Holt-Lunstad, 2017). A contentious or troubled relationship is associated with more mental health problems in mothers and children (Dush & Amato, 2005; Hannighofer *et al.* 2017). To further highlight the importance of the quality of relationship on mental health, prior studies have shown that single people have better mental health outcomes than those who are in an unhappy union (Holt-Lunstad *et al.* 2008). Some authors show that levels of anxiety and depression are considerably higher in single mothers than in married mothers (Crosier *et al.* 2007; Rousou *et al.* 2019), and the Millennium Cohort Study demonstrates that mothers in unstable families (separated or divorced) have worse mental health outcomes than those in stable family units (Baldridge, 2011). Individuals who are in unstable relationships show higher levels of depression and anxiety than those in stable relationships (Cairney *et al.* 2003; Hannighofer *et al.* 2017), which supports the view that positive interaction with partners or a spouse reduces the risk of depression and anxiety (Santini *et al.* 2015). Research indicates that marital status differences in mental health are greatest when the comparison group is the divorced or widowed and smaller or nonsignificant in comparison to the never married, suggesting a more nuanced effect of marriage on mental health outcomes such as anxiety and depression (Cairney & Krause, 2005; Williams & Carlson, 2012).

The impact of relationships and relationship status on anxiety and depression during a pandemic, such as COVID-19, is less studied, and most of the studies in this area involve Asian cohorts. The predominance of studies comprising primarily Asians or set in Asian countries may limit generalizability to other countries and races; hence, the need for a study set in, and comprised of samples from, a different jurisdiction. The evidence varies for studies published to date. Hawryluck *et al.* (2004) found no relationship between marital status and psychological outcomes in quarantine during a crisis, and Wang *et al.* (2020), in examining the psychological responses during the initial phases of the COVID-19 pandemic in China, found that marital status did not significantly impact depression and anxiety scores. However, Tan and colleagues (2020) in their study of the immediate mental health status of the Chinese workforce during the COVID-19 pandemic found that respondents who were divorced, separated, or widowed had higher impact of event, stress, anxiety, and depression scores than individuals who were single; and the married group had lower scores. This study differs from others in that it examined mental health issues in subjects returning to work in a pandemic. The added strain of returning to work in a pandemic and the attendant stress of finding appropriate childcare may have affected responses; while this was not explicitly explored in the study, it does deserve further examination.

The present study examines the impact of relationship status on reports of stress, anxiety, and depression during the COVID-19 pandemic in a Canadian cohort. It aims to add to the literature in this area, which to the best of our knowledge is limited. Most of the literature in this area arises from Asia and may therefore not be generalizable to Canadian subjects; this in turn limits the evidence available for channelling appropriate resources and treatments to those who might need it. Findings may help to provide individuals in at-risk relationship status group with additional mental health supports/services during this and future pandemics.

## Method

A cross-sectional survey was used to explore mean differences in perceived stress, anxiety, and depression symptom scores according to the relationship status of Text4Hope subscribers.

### Recruitment

The study recruitment procedures and sample size estimations have been described in the published study protocol (Agyapong *et al.* 2020). An online survey link was sent to subscribers to the Text4Hope program, a daily supportive text message service, launched by Alberta Health Services on March 23, 2020 to help Albertans cope with the mental health effects of the COVID-19 pandemic. In addition to demographic information, we assessed clinical characteristics using validated scales for self-reported symptoms, including the Perceived Stress Scale (PSS), the Generalized Anxiety Disorder 7-item (GAD-7) scale, and the Patient Health Questionnaire-9 (PHQ-9). The PSS is a validated 10-item questionnaire used to assess the self-reported level of stress in the previous 1 month by assessing thoughts and feelings. Each item on the scale is scored between 0 (never) to 5 (very often). Higher scores on the scale indicates higher levels of stress (Cohen *et al.* 1983). The GAD-7 is a validated 7-item questionnaire used to assess the self-reported levels of anxiety in respondents in the 2 weeks prior to assessment (Spitzer *et al.* 2006). It is based on DSM-IV-TR symptoms of anxiety. Each item on the scale is scored between 0 (not at all) and 4 (nearly every day).

The PHQ-9 is a 9-item validated instrument used to diagnose and measure the severity of depression in general medical and mental health settings (Kroenke *et al.* 2001); it is the major depression module of the full PHQ. Each of the nine items on the questionnaire is scored between 0 (not at all) and 3 (nearly every day). It may be used to plan and monitor treatment of depression.

### Sample size estimation

Based on a provincial population estimate of approximately 4.3 million, the necessary sample size to generate prevalence estimates was 4157, assuming a 99% confidence level and 2% error. Previous research employing similar methodology in Alberta generated a 20% response rate (Agyapong *et al.* 2016). Therefore, we aimed to extract and analyze data after obtaining a minimum recruited sample of 20 785 Text4Hope subscribers.

### Data analysis

Data analysis was undertaken using the IBM Statistical Package for Social Sciences (SPSS) Statistics for Windows, version 26 (Fortuna *et al.* 2018). One-way analysis of variance (ANOVA) with two-tailed significance (*p* < 0.05) was performed to assess the statistical differences between relationship status and corresponding mean scores on the PSS, the GAD-7, and the PHQ-9. For variables which did not violate the assumptions of homogeneity of variance in the mean scores on the ANOVA test, we performed a Tukey’s *post hoc* test to determine if there were statistically significant differences in the mean scores of the various clinical measures between the different relationship status groupings. For variables which violated the homogeneity of variance assumption, we determined if there were statistically significant differences for the mean scores for the various clinical measures between the different relationship status groupings using the Welch F test and a Games-Howell *post hoc* test (as these tests do not require groups to have equal standard deviations).

## Results

Of the 44·992 subscribers who joined Text4Hope in the first 6 weeks, 8267 responded to the online survey invitation, yielding a 19.4% response rate. Of the 8267 respondents, 5799 (70.1%) identified as either married, cohabiting, or partnered, 618 (7.5%) identified as either separated or divorced, 136 (1.6%) identified as widowed, 1541 (18.6%) identified as single, 95 (1.1%) identified as “other,” and 78 (0.9%) did not identify their relationship status.

The other demographic characteristics of the respondents are as shown in Table 1.

**Table 1.** Distribution of demographic characteristics of respondents by relationship status

Variables	Married/cohabiting/partnered N (%)	Separated/divorced N (%)	Widowed N (%)	Single N (%)	Other N (%)	Overall N (%)
Gender						
Male	691 (70.60)	52 (5.30)	8 (0.80)	222 (22.70)	6 (0.60)	979 (12.00)
Female	5055 (71.20)	565 (8.00)	125 (1.80)	1287 (18.10)	72 (1.00)	7104 (86.90)
Other	45 (48.90)	1 (1.10)	1 (1.10)	29 (31.50)	16 (17.40)	92 (1.10)
Age						
≤25	455 (50.30)	11 (1.20)	0 (0.00)	426 (47.10)	13 (1.40)	905 (11.30)
26–40	2251 (76.70)	111 (3.80)	5 (0.20)	545 (18.60)	24 (0.80)	2936 (36.60)
41–60	2513 (73.50)	383 (11.20)	49 (1.40)	445 (13.0)0	31 (0.90)	3421 (42.60)
>60	487 (64.10)	103 (13.60)	75 (9.90)	83 (10.90)	12 (1.60)	760 (9.50)
Ethnicity						
Caucasian	4820 (72.10)	533 (8.00)	117 (1.80)	1167 (17.50)	47 (0.70)	6684 (82.00)
Indigenous	178 (58.90)	25 (8.30)	4 (1.30)	90 (29.80)	5 (1.70)	302 (3.70)
Asian	276 (68.30)	14 (3.50)	1 (0.20)	110 (27.20)	3 (0.70)	404 (5.00)
Other	499 (65.80)	43 (5.70)	10 (1.30)	168 (22.20)	38 (5.00)	758 (9.30)
Education						
Less than high school diploma	114 (34.90)	23 (7.00)	4 (1.20)	177 (54.10)	9 (2.80)	327 (4.00)
High school diploma	532 (65.80)	58 (7.20)	21 (2.60)	190 (23.50)	8 (1.00)	809 (9.90)
Post-secondary education	5097 (73.40)	534 (7.70)	105 (1.50)	1158 (16.70)	53 (0.80)	6947 (85.00)
Other education	47 (51.60)	2 (2.20)	4 (4.40)	13 (14.30)	25(27.50)	91 (1.10)
Employment status						
Employed	4530 (75.70)	443 (7.40)	45 (0.80)	924 (15.40)	40 (0.70)	5982 (73.20)
Unemployed	534 (56.00)	102 (10.70)	24 (2.50)	282 (29.60)	12 (1.30)	954 (11.70)
Retired	384 (68.80)	53 (9.50)	62 (11.10)	54 (9.70)	5 (0.900)	558 (6.80)
Students	204 (45.40)	7 (1.60)	0 (0.00)	233 (51.90)	5 (1.10)	449 (5.50)
Other	137 (58.50)	13 (5.60)	5 (2.10)	46 (19.70)	33 (14.10)	234 (2.90)
Housing status						
Own home	4314 (81.80)	354 (6.70)	106 (2.00)	475 (9.00)	28 (0.50)	5277 (65.7)
Living with family	300 (38.10)	36 (4.60)	2 (0.30)	435 (55.30)	14 (1.80)	787 (9.8)
Renting	1031 (54.80)	214 (11.40)	20 (1.10)	589 (31.30)	27 (1.40)	1881 (23.4)
Other	33 (40.70)	5 (6.20)	2 (2.50)	18 (22.20)	23 (28.40)	81 (1.0)

As demonstrated in Table 1, the majority of respondents were female, (*n* = 7104, 86.9%), were Caucasians (*n* = 6684, 82.0%), had post-secondary education (*n* = 6947, 85.0%), were employed (*n* = 5982, 73.2%), were married, cohabiting, or partnered (*n* = 5799, 70.1%), and owned their own home (*n* = 5277, 65.7%). The mean scores for all the respondents were 20.79 (s.d. = 6.83, *n* = 7589) on the PSS, 9.68 (s.d. = 5.87, *n* = 6944) on the GAD-7 scale, and 9.43 (s.d. = 6.29, *n* = 7082) on the PHQ-9 scale.

Table 2 presents the means and standard deviations for the PSS, GAD-7, and PHQ-9 in relation to the various relationship status groups.

**Table 2.** Mean scores on the GAD-7 scale, PHQ-9 scale, and PSS by relationship status

Scale	Self-reported relationship status	N	Mean	Std. deviation	Std. error	95% confidence interval for mean	
						Lower bound	Upper bound
GAD-7 total score	Married/cohabiting/partnered	4942	9.60	5.80	0.10	9.40	9.80
	Separated/divorced	539	9.30	6.00	0.30	8.80	9.80
	Widowed	117	7.40	5.53	0.50	6.40	8.40
	Single	1270	10.40	5.90	0.10	10.00	10.70
	Other	66	9.10	5.90	0.70	7.70	10.60
	Total	6934	9.70	5.80	0.10	9.50	9.80
PHQ-9 total score	Married/cohabiting/partnered	5034	8.90	6.00	0.10	8.70	9.000
	Separated/divorced	549	10.40	6.70	0.30	9.80	10.90
	Widowed	120	7.90	6.20	0.60	6.80	9.10
	Single	1302	11.30	6.70	0.10	10.90	11.70
	Other	67	9.40	7.16	0.90	7.60	11.10
	Total	7072	9.40	6.29	0.10	9.30	9.60
PSS total score	Married/cohabiting/partnered	5378	20.40	6.65	0.10	20.20	20.60
	Separated/divorced	582	20.90	7.17	0.30	20.40	21.50
	Widowed	129	17.90	6.90	0.60	16.70	19.10
	Single	1410	22.40	7.00	0.20	22.00	22.80
	Other	78	21.60	7.20	0.800	19.90	23.20
	Total	7577	20.80	6.80	0.10	20.60	20.90

GAD-7, Generalized Anxiety Disorder 7 scale; PHQ, Patient Health Questionnaire; PSS, Perceived Stress Scale.

Table 2 shows that the mean scores on the PSS, GAD-7, and PHQ-9 were highest among those who were single and lowest among those who were widowed. Respondents who were either married, cohabiting, or partnered and those who were either separated or divorced had similar mean scores on the PSS and GAD-7. However, respondents who were either separated or divorced had a higher mean score on the PHQ-9 than respondents who were either married, cohabiting, or partnered.

Table 3 represents the results of the one-way ANOVA comparing the sums of squares between and within relationship status groups for the PSS, GAD-7, and PHQ-9.

**Table 3.** One way ANOVA comparing sums of squares between and within groups

		Sum of squares	DF	Mean square	F statistic	Sig.
GAD-7 total score	Between groups	1350.20	4	337.60	9.90	< 0.01*
	Within groups	237·190.60	6929	34.20		
	Total	238·540.80	6933			
PHQ-9 total score	Between groups	7087.10	4	1771.80	45.90	< 0.01*
	Within groups	272·566.90	7067	38.60		
	Total	279·654.50	7071			
PSS total score	Between groups	5590.10	4	1397.50	30.50	< 0.01*
	Within groups	347·437.80	7572	45.90		
	Total	353·027.90	7576			

GAD-7, Generalized Anxiety Disorder 7 scale; PHQ, Patient Health Questionnaire; PSS, Perceived Stress Scale; DF, degree of freedom.

* *p* < 0.05.

Table 3 demonstrates that there were statistically significant differences between and within relationship status groups for scores on the PSS (F = 30.46, *p* < 0.01), GAD-7 (F = 9.86, *p* < 0.01), and PHQ-9 (F = 45.94, *p* < 0.01). The Levene statistic test of homogeneity of variances suggested no violation of the assumption of equality of means for the GAD-7 (*p* > 0.05), and thus, a Tukey’s *post hoc* test was conducted to determine statistically significant differences in the mean scores between the different relationship status groups as presented in Table 4. However, the Levene statistic test of homogeneity of variances suggested there was a violation of the assumption of equality of means for the PSS and PHQ-9 (*p* < 0.05). Consequently, a Welch F test and a Games-Howell *post hoc* test were carried out to determine statistically significant differences in the mean scores on the two scales between the different relationship status groups. The Welsh F tests were statistically significant in each case, which confirms that the differences between the groups in terms of their mean PSS and PHQ-9 scores were statistically significant. Results of the Games-Howell *post hoc* test is presented in Table 5.

**Table 4.** Tukey HSD *post hoc* multiple comparison

Dependent variable	(I) Relationship	(J) Relationship	Mean difference (I–J)	Std. error	*p*-Value*	95% confidence interval	
						Lower bound	Upper bound
GAD-7 total score	Married/cohabiting/partnered	Separated/divorced	0.30	0.27	0.78	−0.42	1.03
		Widowed	2.23	0.55	<0.01*	0.73	3.72
		Single	−0.77	0.18	<0.01*	−1.27	−0.26
		Other	0.47	0.73	0.97	−1.51	2.45
	Separated/divorced	Married/cohabiting/partnered	−0.30	0.27	0.78	−1.03	0.42
		Widowed	1.92	0.60	0.01*	0.30	3.55
		Single	−1.07	0.30	<0.01*	−1.89	−0.25
		Other	0.16	0.76	1.00	−1.92	2.25
	Widowed	Married/cohabiting/partnered	−2.23	0.55	<0.01*	−3.72	−0.73
		Separated/divorced	−1.92	0.60	0.01*	−3.55	−0.30
		Single	−2.99	0.57	<0.01*	−4.54	−1.45
		Other	−1.76	0.90	0.29	−4.22	0.70
	Single	Married/cohabiting/partnered	0.77	0.18	<0.01*	0.26	1.27
		Separated/divorced	1.07	0.30	<0.01*	0.25	1.89
		Widowed	2.99	0.57	<0.01*	1.45	4.54
		Other	1.23	0.74	0.45	−0.78	3.25
	Other	Married/cohabiting/partnered	−0.47	0.73	0.97	−2.45	1.51
		Separated/divorced	−0.16	0.76	0.10	−2.25	1.92
		Widowed	1.76	0.90	0.29	−0.70	4.22
		Single	−1.23	0.74	0.45	−3.25	0.78

GAD-7, Generalized Anxiety Disorder-7 item scale.

* *p* < 0.05.

**Table 5.** Games-Howell *post hoc* multiple comparison

Dependent variable	(I) Relationship	(J) Relationship	Mean difference (I–J)	Std. error	Sig.	95% confidence interval	
						Lower bound	Upper bound
PHQ-9 total scor**e**	Married/cohabiting/partnered	Separated/divorced	−1.52	0.30	<0.01*	−2.33	−0.70
		Widowed	0.93	0.57	0.48	−0.65	2.50
		Single	−2.47	0.20	<0.01*	−3.02	−1.91
		Other	−0.52	0.88	0.976	−2.98	1.94
	Separated/divorced	Married/cohabiting/partnered	1.52	0.30	<0.01*	0.70	2.33
		Widowed	2.44	0.63	0.01*	0.70	4.18
		Single	−0.95	0.34	0.04*	−1.88	−0.03
		Other	0.99	0.92	0.81	−1.57	3.56
	Widowed	Married/cohabiting/partnered	−0.93	0.57	0.48	−2.50	0.65
		Separated/divorced	−2.44	0.63	0.01*	−4.18	−0.70
		Single	−3.40	0.59	<0.01*	−5.03	−1.76
		Other	−1.45	1.04	0.64	−4.33	1.43
	Single	Married/cohabiting/partnered	2.47	0.20	<0.01*	1.91	3.02
		Separated/divorced	0.954	0.34	0.04*	0.03	1.88
		Widowed	3.40	0.59	<0.01*	1.76	5.03
		Other	1.95	0.89	0.20	−0.55	4.45
	Other	Married/cohabiting/partnered	0.519	0.88	0.98	−1.94	2.98
		Separated/divorced	−0.99	0.92	0.81	−3.56	1.57
		Widowed	1.45	1.04	0.64	−1.43	4.33
		Single	−1.95	0.89	0.20	−4.45	0.55
PSS total score	Married/cohabiting/partnered	Separated/divorced	−0.54	0.31	0.42	−1.39	0.31
		Widowed	2.49	0.61	0.01*	0.80	4.19
		Single	−1.99	0.21	<0.01*	−2.57	−1.43
		Other	−1.18	0.83	0.62	−3.50	1.15
	Separated/divorced	Married/cohabiting/partnered	0.537	0.31	0.42	−0.31	1.39
		Widowed	3.03	0.68	<0.01*	1.17	4.89
		Single	−1.46	0.35	<0.01*	−2.42	−0.50
		Other	−0.64	0.88	0.95	−3.08	1.80
	Widowed	Married/cohabiting/partnered	−2.49	0.61	0.01*	−4.19	−0.80
		Separated/divorced	−3.03	0.68	<0.01*	−4.89	−1.17
		Single	−4.49	0.63	<0.01*	−6.24	−2.74
		Other	−3.67	1.03	<0.01*	−6.50	−0.84
	Single	Married/cohabiting/partnered	1.99	0.21	<0.01*	1.43	2.57
		Separated/divorced	1.46	0.35	<0.01*	0.50	2.42
		Widowed	4.49	0.63	<0.01*	2.74	6.24
		Other	0.82	0.85	0.87	−1.54	3.18
	Other	Married/cohabiting/partnered	1.18	0.83	0.62	−1.15	3.50
		Separated/divorced	0.64	0.88	0.95	−1.80	3.08
		Widowed	3.67	1.03	<0.01*	0.84	6.50
		Single	−0.82	0.85	0.87	−3.18	1.54

PHQ, Patient Health Questionnaire; PSS, Perceived Stress Scale.

* The mean difference is significant at the 0.05 level.

Table 4 suggests that respondents who identified as either married, cohabiting, or partnered had significantly higher mean scores on the GAD-7 compared to respondents who identified as widowed (mean difference = 2.23, 95% CI = 0.73–3.72, and *p* < 0.01) but not respondents who identified as separated or divorced or other (*p* > 0.05). On the other hand, respondents who were either married, cohabiting, or partnered were significantly more likely to have a lower mean score on the GAD-7 compared to respondents who were single (mean difference = 0.77, 95% CI = −1.27–−0.26, *p* < 0.01). Table 4 also suggests that respondents who were single had a significantly higher mean score on the GAD-7 compared to respondents who were either separated or divorced (mean difference = 1.07, 95% CI = 0.25–1.89, *p* < 0.01) and respondents who were widowed (mean difference = 2.99, 95% CI = 1.45–4.54, *p* < 0.01). Finally, respondents who were either separated or divorced had a significantly higher mean score on the GAD-7 compared to respondents who were widowed (mean difference = 1.92, 95% CI = 0.3–3.55, *p* < 0.01).

Table 5 shows that respondents who identified as either married, cohabiting, or partnered had significantly lower mean scores on the PHQ-9 compared to respondents who identified as separated or divorced (mean difference = −1.52, 95% CI = −2.33–−0.7, *p* < 0.01) and respondents who identified as single (mean difference = −2.47, 95% CI = −3.02–−1.91, *p* < 0.01), but not respondents who identified as either widowed or other (*p* > 0.05). On the other hand, respondents who were single had significantly higher mean scores on the PHQ-9 compared to respondents who were separated or divorced (mean difference = 0.95, 95% CI = 0.03–1.88, *p* = 0.04) and those who were widowed (mean difference = 3.40, 95% CI = 1.76–5.03, *p* < 0.01). Finally, respondents who identified as separated or divorced had a significantly higher mean score on the PHQ-9 compared to respondents who were widowed (mean difference = 2.44, 95% CI = 0.70–4.18, *p* < 0.01).

Table 5 also shows that respondents who identified as either married, cohabiting, or partnered had significantly higher mean scores on the PSS compared to respondents who identified as widowed (mean difference = 2.94, 95% CI = 0.8–4.19, *p* < 0.01) and a significantly lower mean score than respondents who identified single (mean difference = −1.99, 95% CI = −2.57–−1.43, *p* < 0.01), but not respondents who identified as either widowed or other (*p* > 0.05). On the other hand, respondents who were single had significantly higher mean scores on the PSS compared to respondents who were separated or divorced (mean difference = 1.46, 95% CI = 0.50–2.42, *p* < 0.01) and those who were widowed (mean difference = 4.49, 95% CI = 2.74–6.24, *p* < 0.01). Finally, respondents who identified as separated or divorced had a significantly higher mean score on the PSS compared to respondents who were widowed (mean difference =3.03, 95% CI = 1.17–4.89, *p* < 0.01).

## Discussion

This is the first study in Canada to specifically examine the impact of relationship status on measures of self-reported stress, anxiety, and depression during the COVID-19 pandemic. The majority of the participants in this study were Caucasian (*n* = 6684, 82%), female (*n* = 7104, 86.9%), aged between 26 and 60 years (*n* = 6357, 79.2%), with post-secondary school education (*n* = 6947, 85.0%), employed (*n* = 5982, 73.2%), and living in their own home (*n* = 5277, 65.7%). These figures (see Table 1) suggest a degree of socioeconomic stability within the sample, prior to COVID-19 pandemic onset.

The majority of individuals in the cohort identified as married, cohabiting, or partnered (*n* = 5799, 70.1%). This is comparable to the findings of Wang *et al.* (Wang *et al.* 2020) who surveyed the general public in mainland China in the early weeks of the COVID-19 pandemic and found that 76.4% of their participants reported being married. We found a consistent trend of higher mean scores on the PSS, GAD-7, and PHQ-9 in responders who identified as single compared to those who were married, cohabiting, or partnered, or indeed in any other relationship category. This suggests that being in a relationship of some sort mitigates the risk of developing symptoms of anxiety, depression, or stress during the COVID-19 pandemic. This is not surprising as having someone around helps to provide a means of socializing particularly with the restrictions in socializing put in place at some points during the pandemic. Individuals who identified as separated or divorced also reported higher mean scores across measures compared to those who were married, partnered, or cohabiting, although this difference was more distinct for PHQ-9 than GAD-7 or PSS. Individuals who were widowed consistently reported low levels of anxiety and depression compared to other groups; this was not in alignment with the finding of Tan *et al.* (2020). It is possible that having been through the loss of a partner and its attendant grief, these individuals may have developed the resilience to help them cope with the pandemic.

Social isolation and loneliness have been identified as major adverse consequences of the COVID-19 pandemic (Menon *et al.* 2015). Other studies have reported that when people are isolated or lonely, they become significantly more vulnerable to anxiety, depression, deliberate self-harm, and suicide (Elovainio *et al.* 2017; Matthews *et al.* 2019; Nkire *et al.* 2021). Single individuals are certainly more likely to feel the effects of loneliness and isolation more than the married (Matthews *et al.* 2019), and this may explain the consistently higher levels of stress, anxiety, and depression among the single compared to other subgroups within this cohort. Available evidence suggests that measures aimed at reducing loneliness and promoting connectedness can be protective against emotional problems, deliberate self-harm, and completed suicide (Stack, 1988; O’Connor & Kirtley, 2018). While this study did not specifically ask respondents about loneliness, we hypothesize that single individuals were more likely to be lonely and socially isolated in quarantine and self-isolation, and future studies may explore this hypothesis. As such, results from the present study suggests a need for early interventions that are targeted at people who are single, with a view to preventing or mitigating mental health consequences of the COVID-19 pandemic or future crisis situations.

Wang *et al.* in their study of a Chinese cohort demonstrated that 75.2% of respondents reported experiencing some worry about the prospects of a family member becoming infected with the COVID-19 virus (Wang *et al.* 2020). This same study found that increased levels of concern that a family member would become infected was significantly associated with depression, anxiety, and stress (DAS) subscale scores (B = 0.50, 95% CI = 0.04–0.96); the present study showed that respondents who are married, cohabiting, or partnered had higher mean anxiety scores compared to widowed. This finding may be a reflection of the concerns about infecting a partner. It merits further exploration.

This study has several strengths and limitations. The use of anonymous online surveys ensures an element of blinding and mitigated the risk of bias on the part of a potential assessor or bias on the part of the respondent; it also ensured anonymity of the individuals completing the survey. On the downside, the nature and quality of relationships in the different subgroups was not explored; as well, the survey did not clarify whether people who identified as single had other close and reliable social networks such as supportive roommate situations which could well impact their coping abilities and sense of distress. The use of a self-report survey poses a limitation in the actual definition of illness, as assessment by trained mental health clinicians administering the survey may yield potentially differing results. Generalization is limited by the study sample being based primarily in Alberta Canada, and the respondents are individuals who are specifically enrolling into a service to receive anxiety and stress support. Furthermore, we were unable to measure respondents’ pre-COVID-19 baseline scores for stress, anxiety, and depression. The relatively low response rate may open the study to nonresponse bias. However, the study sample was greater than the projected sample size of 4157 needed to accurately estimate prevalence rates of mental health conditions in an Alberta population with a 99% confidence and a 2% margin of error. The use of an anonymous recruitment process also meant that we were unable to compare how responders differed from nonresponders both clinically and demographically although our representative sample suggest the two groups would have similar characteristics. In addition, while a one-way ANOVA allowed for comparison of the stress, anxiety, and depression levels between all the relationship groups as a strength, it did not take into account potential confounding factors such as sex, age, ethnicity, employment, and education status, which is a limitation. The impact of these confounding factors could be assessed using regression models, although such models would also have the limitation of not allowing for a comparison of the stress, anxiety, and depression between all relationship status categories. Notwithstanding these limitations, the findings from this study shed light on the effects of relationship status on reports of stress, anxiety, and depression in the early stages of the COVID-19 pandemic. The nature of recruitment into this study allowed for increased respondent diversity and generalizability as well as affording us the opportunity to investigate some demographic predictors. The findings are in alignment with existing literature from other geographical areas.

Results from this study suggests that being single and separated or divorced are risk factors for more severe outcome stress, anxiety, and depression scores specifically during the COVID-19 pandemic. Services aimed at providing mental health supports during pandemics should consider allocating more resources to supporting these particular groups of people. For example, supportive text messages are independent of geographic location, are free to the end users, do not require expensive data plans, and can reach thousands of people simultaneously (Agyapong *et al.* 2011, 2016). Previous research has reported that daily supportive text messages are effective in reducing depressive symptoms as well as supporting individuals with problem drinking (Agyapong *et al.* 2012, 2013a, 2015, 2017, 2018; Hartnett *et al.* 2017; O’Reilly *et al.* 2019). High user satisfaction has also been reported (Agyapong *et al.* 2013a, 2016). Therefore, innovative and cost-effective interventions such as the Text4Hope program (Agyapong 2020; Agyapong *et al.* 2020) could be useful particularly to a single and separated or divorced individual who seem to be most impacted psychologically during the COVID-19 pandemic.
